# Comparative analysis of bone metabolism and inflammatory markers in tibiofibular fractures following closed reduction and fixation: A retrospective study

**DOI:** 10.5937/jomb0-55571

**Published:** 2025-07-04

**Authors:** Lingfeng Li

**Affiliations:** 1 Fifth People's Hospital, Fudan University, Happy Center Hospital Shanghai, Shanghai, China

**Keywords:** serum BGP, BALP, P1NP, b-CTX, CRP, IL-6, IL-1b, TNF-a, tibiofibular fracture, closed reduction, internal fixation, external fixation, bone metabolism, inflammation, serum BGP, BALP, P1NP, b-CTX, CRP, IL-6, IL-1b, TNF-a, tibiofibularni prelom, zatvorena redukcija, interna fiksacija, eksterna fiksacija, metabolizam kostiju, inflamacija

## Abstract

**Background:**

This study aimed to evaluate the effectiveness and safety of closed reduction and internal fixation (CRIF) versus closed reduction and external fixation (CREF) in treating tibiofibular fractures, focusing on their impact on bone metabolism and inflammatory responses.

**Methods:**

A retrospective analysis was conducted on the clinical data of 95 patients with tibiofibular fractures, categorised into the CRIF group (CRIFG) and the CREF group (CREFG). Clinical efficacy, Visual Analogue Scale (VAS) scores, serum bone metabolism markers, serum inflammatory cytokines, Generic Quality of Life Inventory-74 (GQOLI-74) scores, and adverse reactions (AR) were compared between the groups.

**Results:**

The total clinical efficacy rates were 80.49% (33/41) in the CRIFG and 85.19% (46/54) in the CREFG (P>0.05). Compared to CRIFG, the CREFG group exhibited significantly lower VAS scores and higher GQOLI-74 scores across all dimensions. Additionally, the CREFG group showed increased levels of serum osteocalcin (BGP), bone alkaline phosphatase (BALP), and N-terminal propeptide of type 1 procollagen (P1NP), along with decreased levels of type I collagen carboxy-terminal peptide b special sequence (b-CTX). Inflammatory markers, including C-reactive protein (CRP), interleukin-6 (IL-6), IL-1b, and tumour necrosis factor-a (TNF-a), were significantly lower in the CREFG group. The total AR rate was also lower in CREFG (18.52% vs. 31.71%, P<0.05).

**Conclusions:**

Compared to CRIF, CREF treatment is more effective in reducing pain, enhancing bone metabolism, alleviating inflammatory responses, and improving the overall quality of life (QoL) in patients with tibiofibular fractures.

## Abstract (sr)

**Uvod:**

Cilj ove studije bio je da se oceni efikasnost i bezbednost zatvorene redukcije i interne fiksacije (CRIF) u poređenju sa zatvorenom redukcijom i eksternom fiksacijom (CREF) u lečenju tibiofibularnih preloma, sa fokusom na njihov uticaj na metabolizam kostiju i inflamatorne odgovore.

**Metode:**

Sprovedena je retrospektivna analiza kliničkih podataka 95 pacijenata sa tibiofibularnim prelomima, podeljenih u grupu CRIF (CRIFG) i grupu CREF (CREFG). Upoređivane su kliničke efikasnosti, VAS skorovi (Vizuelno analogna skala), markeri metabolizma kostiju u serumu, serumski inflamatorni citokini, skorovi Generalnog inventara kvaliteta života-74 (GQOLI-74) i neželjene reakcije (AR) između grupa.

**Rezultati:**

Ukupna klinička efikasnost je bila 80,49% (33/41) u grupi CRIFG i 85,19% (46/54) u grupi CREFG (P>0,05). U poređenju sa grupom CRIFG, grupa CREFG je imala značajno niže VAS skore i više GQOLI-74 skorove u svim dimenzijama. Dodatno, grupa CREFG je pokazala povećane nivoe serumskog osteokalcina (BGP), alkalne fosfataze kostiju (BALP) i N-terminalnog propeptida tipa 1 prokolagena (P1NP), uz smanjenje nivoa karboksiterminalnog peptida tipa I kolagena b specifične sekvence (b-CTX). Inflamatorni markeri, uključujući C-reaktivni protein (CRP), interleukin-6 (IL-6), IL-1b i faktor nekroze tumora (TNF-a), bili su značajno niži u grupi CREFG. Ukupna stopa AR bila je takođe niža u grupi CREFG (18,52% naspram 31,71%, P<0,05).

**Zaključak:**

U poređenju sa CRIF, CREF tretman je efikasniji u smanjenju bola, poboljšanju metabolizma kostiju, ublažavanju inflamatornih odgovora i poboljšanju ukupnog kvaliteta života (QoL) kod pacijenata sa tibiofibularnim prelomima.

## Introduction

The tibia and fibula are critical in maintaining ankle joint stability, weight-bearing, and locomotion.Due to their structural importance, these bones are highly susceptible to fractures resulting from direct or indirect trauma, including high-energy impacts, falls, and sports injuries. Tibiofibular fractures are often associated with significant pain, deformity, and impaired mobility, which necessitate timely and appropriate treatment to restore function and prevent complications [1]. Proper reduction and fixation are essential to achieving anatomical alignment, promoting bone healing, and preventing long-term disability [2].

Several surgical approaches are available for managing tibiofibular fractures, including plate internal fixation, intramedullary nailing, and external fixation [3] [4]. Among these, closed reduction and internal fixation (CRIF) using intramedullary nails is widely preferred due to its biomechanical stability and ability to maintain bone alignment. However, CRIF is associated with soft tissue trauma and postoperative complications, such as infection, delayed union, and nonunion, particularly in patients with extensive soft tissue damage [5].

Alternatively, closed reduction and external fixation (CREF) has been proposed as a viable alternative, particularly in severe soft tissue compromise cases. External fixation provides stable alignment with minimal surgical trauma, reduces the risk of postoperative infection, and facilitates early mobilisation. Furthermore, CREF has been reported to influence bone metabolism and inflammatory responses, which play crucial roles in fracture healing and patient recovery [6].

Despite the advantages of both CRIF and CREF, there is limited comparative research focusing on their effects on bone metabolism and inflammatory responses in tibiofibular fractures. Therefore, this study aims to compare the clinical outcomes of CRIF and CREF, specifically analysing their impact on serum bone metabolism markers (osteocalcin [BGP], bone alkaline phosphatase [BALP], N-terminal propeptide of type 1 procollagen [P1NP], and β-CrossLaps [β-CTX]) and inflammatory cytokines (C-reactive protein [CRP], interleukin-6 [IL-6], interleukin-1β [IL-1β], and tumour necrosis factor-α [TNF-α]). The findings will contribute to optimising treatment strategies for tibiofibular fractures and improving patient prognosis.

## Materials and methods

### Subjects

A retrospective collection of clinical data from 95 cases of tibiofibular fracture patients who underwent closed reduction treatment at Happy Center Hospital Shanghai Fifth People’s Hospital from October 2022 to March 2024 was conducted. Inclusion criteria: people conforming to the diagnostic criteria for tibiofibular fracture in *Practical Orthopedics* [7] and being closed, simple, and fresh fractures; complete clinical data. Exclusion criteria: open, comminuted, pathological fractures or fractures combined with other parts; severe infection, uncontrolled diabetes, immune diseases, or visible organ dysfunction; mental dysfunction or cognitive impairment; long-term use of anticoagulant drugs. It was approved by the Happy Center Hospital Shanghai Fifth People’s Hospital ethics committee [8] [9] [10].

According to the surgical treatment method, patients were grouped into CRIFG and CREFG, including 41 and 54 patients, respectively. The CRIFG had 22 men and 19 women; age ranged from 20 to 53 (38.3±5.4) years; time from injury to hospital admission: 1.0 to 9.4 (4.8±1.1) h; causes of injury included traffic injuries in 24 cases, falls from a height in 10 cases, heavy object crush injuries in 2 cases, and other factors in 5 cases; fracture sites were on the left side in 20 cases and on the right side in 21 cases. The CREFG had 28 men and 26 women; age ranged from 18 to 56 (39.1±6.0) years; time from injury to hospital admission: 1.1 to 10.5 (4.6±1.5) h; causes of injury included traffic injuries in 28 cases, falls from a height in 13 cases, heavy object crush injuries in 5 cases, and other factors in 8 cases; fracture sites were on the left side in 29 cases and on the right side in 25 cases. No statistically meaningful distinction was noted in the general data of the subjects (*P*>0.05).

### Surgical methods

Patients were positioned supine for closed reduction and internal fixation (CRIF), and standard sterile draping was applied following routine disinfection. Epidural anaesthesia was administered to ensure adequate pain control. A longitudinal incision (approximately 5–6 cm) was made along the patellar ligament, with the tibial tubercle serving as the entry point for medullary cavity preparation. The cavity was drilled and expanded to accommodate an appropriately sized interlocking intramedullary nail. Under C-arm fluoroscopic guidance, the fracture was reduced, and the intramedullary nail was inserted into the distal segment of the tibia. Once the reduction was confirmed, distal locking screws were applied for stabilisation. Postoperative care included prophylactic antibiotics to prevent infection and administering analgesic and anti-inflammatory medications. Early functional rehabilitation was initiated based on the patient’s recovery status [11] [12] [13] [14] [15].

For closed reduction and external fixation (CREF), patients were also placed supine, and routine disinfection was followed by sterile draping. Epidural anaesthesia was administered before manual traction was performed to achieve fracture reduction. The fracture alignment was confirmed using a C-arm fluoroscopic unit. External fixation pins were drilled and inserted at predetermined positions, followed by attaching a pin holder and a crossbar, maintaining a distance of approximately 2 cm from the skin. A fluoroscopic evaluation was repeated to ensure optimal fracture reduction and fixation. Once the alignment was satisfactory, screws were tightened to secure the construct. Postoperatively, antibiotics were administered prophylactically to minimise infection risk and analgesic and anti-inflammatory medications were given as needed. Depending on the patient’s postoperative progress, early mobilisation and functional rehabilitation exercises were encouraged [16] [17] [18].

### Observational indicators

Surgical Outcomes: Perioperative data were recorded, including hospital stay duration, intraoperative blood loss, postoperative swelling duration, and fracture healing time.

Pain Assessment: The Visual Analogue Scale (VAS) was used to assess pain levels at baseline (0 days) and 1, 3, 7, and 14 days postoperatively. The scale ranges from 0 (no pain) to 10 (worst imaginable pain), with higher scores indicating greater pain intensity [8].

Bone Metabolism Markers: Fasting venous blood samples were collected at baseline (0 days) and at 1, 3, 7, and 14 days postoperatively. Samples were centrifuged at 3,000 rpm for 10 minutes to obtain serum, which was analysed for key bone metabolism markers, including osteocalcin (BGP), bone alkaline phosphatase (BALP), N-terminal propeptide of type 1 procollagen (P1NP), and β-CrossLaps (β-CTX). These biomarkers were quantified using enzymelinked immunosorbent assay (ELISA) kits from Beckman Coulter (USA), following the manufacturer’s protocols [12].

Inflammatory Markers: Systemic inflammatory responses were assessed by measuring serum levels of C-reactive protein (CRP), interleukin-6 (IL-6), interleukin-1β (IL-1β), and tumour necrosis factor-α (TNF-α). Blood samples were centrifuged at 3,000 rpm for 10 minutes, and inflammatory markers were analysed using ELISA kits from Beckman Coulter (USA), strictly adhering to the manufacturer’s instructions [19].

Clinical Efficacy Evaluation: Treatment outcomes were evaluated using radiographic imaging and functional assessments. A good outcome was defined as fully restoring limb function, proper anatomical fracture alignment, and satisfactory positioning of fracture ends. An effective outcome indicated partial restoration of function, with at least onethird alignment of the fracture plane and partial fracture end continuity. An ineffective outcome was noted if there was no improvement or worsening limb function, non-union, or local infection. The total clinical efficacy rate was calculated using the formula:

Number of patients with good or effective outcomesTotal number of patients×100%\frac{\text{ Number of patients with good or effective outcomes}}{\text{Total number of patients}} \times 100\%

Quality of Life (QoL) Assessment: Postoperative QoL was assessed using the Generic Quality of LifeInventory-74 (GQOLI-74), which evaluates multiple dimensions, including physical function, psychological well-being, social function, and overall life status. The questionnaire consists of 74 items, each rated on a 5-point scale, with higher scores indicating better quality of life [9].

Adverse Reactions (AR): The occurrence of postoperative complications, including wound infection, chronic oedema, non-union, nerve damage, and secondary fractures, was systematically recorded and analysed to compare the safety profiles of CRIF and CREF.

### Statistical processing

SPSS 23.0 software was employed. Count data were presented by frequency or %, and the X^2^ test was adopted. Quantitative data were presented by mean ± sd, a *t*-test was adopted. A* P*-value of less than 0.05 was considered statistically meaningful.

## Results

### Contrast of surgical indicators

In Table 1, the hospital stay, intraoperative blood loss, postoperative swelling time, and fracture healing time in the CREFG were all visibly less than against the CRIFG (*P*<0.05).

**Table 1:** Contrast of perioperative surgical indicators of the subjects.

Indicators	CRIFG<br>(n=41)	CREFG<br>(n=54)	*P*
Intraoperative<br>blood loss (mL)	133.2±10.1	83.5±6.4	0.000
Postoperative<br>detumescence time (d)	11.6±1.3	7.2±1.1	0.000
Length of<br>hospital stay (d)	12.8±1.9	8.8±1.4	0.000
Fracture healing<br>time (months)	8.5±2.2	6.7±1.6	0.000

### Contrast of pain degree

**Figure 1:**
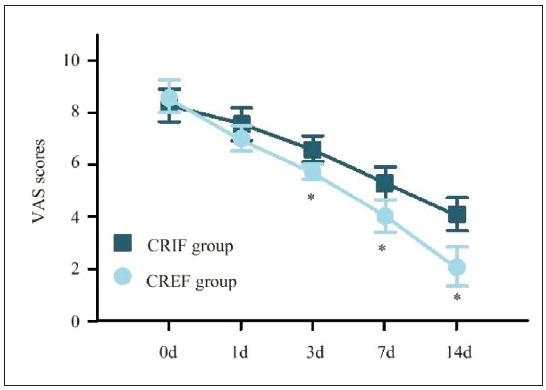
Contrast of VAS scores for subjects at various time points.<br>Note: * as against the CRIFG, P<0.05

In Figure 1, the VAS scores of both the CRIFG and the CREFG gradually decreased with the increase of treatment time. The VAS scores at 3d, 7d, and 14d following remedy in the CREFG were visibly lower as against the CRIFG (*P*<0.05).

### Contrast of bone metabolism

**Figure 2:**
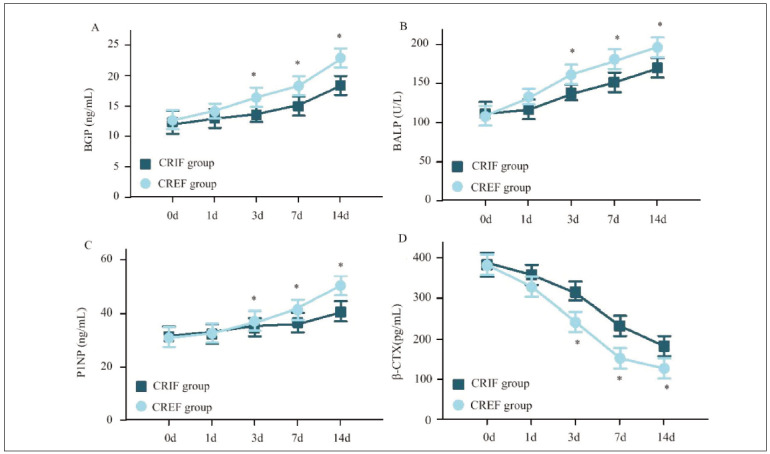
Contrast of serum bone metabolism indicators for subjects at various time points.<br>Note: A: BGP; B: BALP; C: P1NP; D: β-CTX; * as against the CRIFG, P<0.05

In Figure 2, the levels of serum bone metabolism indicators BGP, BALP, and P1NP in both the CRIFG and the CREFG gradually increased with the prolongation of remedy time, while the level of serum β-CTX gradually decreased. At 3d, 7d, and 14d following remedy, GP, BALP, and P1NP were visibly higher, and β-CTX was visibly lower in the CREFG as against the CRIFG (*P*<0.05).

### Contrast of inflammatory response

**Figure 3:**
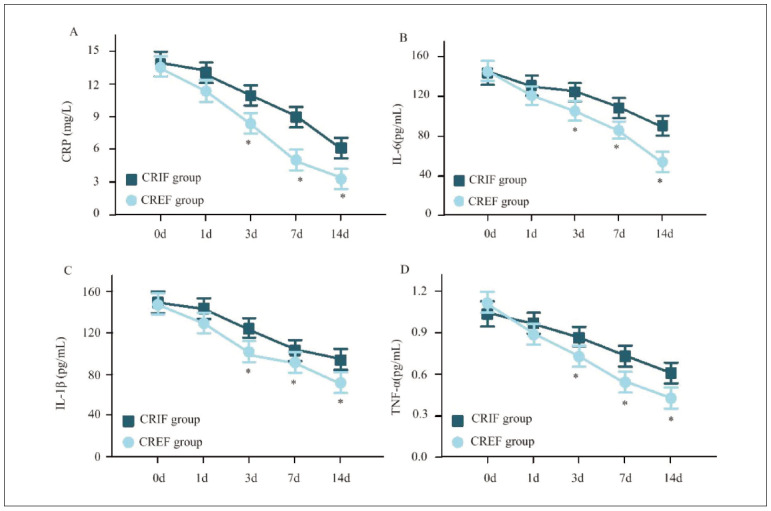
Contrast of serum inflammatory factors for subjects at various time points.<br>Note: A: CRP; B: IL-6; C: IL-1β; D: TNF-α; * as against the CRIFG, P<0.05

In Figure 3, the levels of serum inflammatory factors in both the CRIFG and the CREFG gradually decreased with the increase of remedy time. At 3d, 7d, and 14d following remedy, those in the CREFG were visibly lower as against the CRIFG (*P*<0.05).

### Contrast of clinical response

In Table 2, the total clinical response rates of the CRIFG and the CREFG were 80.49% (33/41) and85.19% (46/54), respectively. No statistically meaningful distinction was noted in the total clinical response rates of the subjects (*P*>0.05).

**Table 2:** Contrast of clinical efficacy in the subjects (n, %).

Efficacy	CRIFG<br>(n=41)	CREFG<br>(n=54)	*P*
Obvious effect	24, 58.54	35, 64.81	
Effective	9, 21.95	11, 20.37	
Invalid	8, 19.51	8, 14.81	
**Total response**	**33, 80.49**	**46, 85.19**	**0.052**

### QoL

In Table 3, the scores for social function, life status, physical function, and psychological function in both the CRIFG and the CREFG were higher following remedy. Following remedy, the scores in the CREFG were markedly superior as against the CRIFG (*P*<0.05).

**Table 3:** Contrast of QoL scores in the subjects. Note: *P* values are statistical values for the contrast of post-remedy scores between CRIFG and CREFG

Items	CRIFG (n=41)		CREFG (n=54)		P
	Before remedy	Following remedy	Before remedy	Following remedy	
Social function	60.8±5.5	74.5±5.6	61.6±4.8	80.8±5.3	0.025
Life status	64.3±6.0	78.9±4.4	64.1±5.9	83.2±4.1	0.016
Physical<br>function	65.2±4.7	78.5±5.3	64.4±5.3	82.7±3.7	0.010
Psychological function	63.8±4.0	79.1±6.6	64.0±3.7	82.6±5.0	0.012

### Contrast of AR

Comparisons were made regarding the occurrence of AR during the remedy period between the CRIFG and the CREFG. In the CRIFG, there were 3 cases of wound infection (7.31%), 1 case of osteomyelitis (2.44%), 5 cases of chronic oedema (12.20%), 2 cases of non-union of bones (4.88%), and 2 cases of nerve damage (4.88%), totalling 13 ARs with an incidence rate of 31.71%. In the CREFG, there were 2 cases of wound infection (3.70%), 4 cases of chronic oedema (7.41%), 2 cases of nonunion of bones (3.70%), 1 case of nerve damage (1.85%), and 1 case of secondary fracture (1.85%), totalling 10 ARs with an incidence rate of 18.52%. The total incidence rate of AR in the CREFG was markedly lower than against the CRIFG (*P*<0.05).

## Discussion

Tibiofibular fractures are common in clinical practice and significantly impact a patient’s mobility and quality of life. The primary goal of treatment is to restore limb stability, ensure proper anatomical alignment, and minimise complications [10] [11]. While closed reduction and internal fixation (CRIF) using interlocking intramedullary nails is widely adopted, closed reduction and external fixation (CREF) has gained attention due to its minimal invasiveness and reduced surgical trauma [12]. In this study, CREF resulted in significantly lower intraoperative blood loss, shorter hospital stays, reduced postoperative swelling time, and faster fracture healing than CRIF (Table 1). These advantages are likely attributed to the minimally invasive nature of CREF, which avoids extensive soft tissue dissection and preserves periosteal blood supply, thereby enhancing fracture healing [14]. However, no statistically significant difference was found in the overall clinical efficacy rates between CRIFG and CREFG (*P*>0.05), aligning with previous findings that suggest both fixation methods effectively facilitate fracture healing but through different mechanisms [13]. CRIF provides internal stabilisation, while CREF offers rigid external support, reducing movement at the fracture site while allowing early weight-bearing and mobilisation [14].

Bone metabolism plays a crucial role in fracture healing by balancing bone formation and resorption. Several serum biomarkers provide objective insights into this process. Osteocalcin (BGP) is a non-collagenous protein synthesised by osteoblasts and is considered a reliable marker of bone formation [16]. Increased BGP levels indicate enhanced osteoblast activity and bone mineralisation. In this study, BGP levels were significantly higher in CREFG than in CRIFG at 3d, 7d, and 14d postoperatively (*P*<0.05), suggesting that CREF provides a more favourable environment for osteoblast activity and bone regeneration. Bone alkaline phosphatase (BALP), another key bone formation marker, facilitates the mineralisation of newly formed bone matrix [17]. Similar to BGP, BALP levels were significantly higher in CREFG compared to CRIFG at multiple time points (*P*<0.05), indicating a more pronounced osteogenic response. N-terminal propeptide of type 1 procollagen (P1NP), an early marker of collagen synthesis and bone formation, reflects osteoblast activity in producing new bone tissue [18]. Elevated P1NP levels in CREFG at 3d, 7d, and 14d (*P*<0.05) suggest accelerated bone matrix formation and enhanced fracture healing. In contrast, β-CrossLaps (β-CTX), a marker of bone resorption released during collagen degradation, was significantly lower in CREFG than in CRIFG at all postoperative time points (*P*<0.05), reflecting decreased osteoclast activity and reduced bone turnover [19] [20] [21]. These findings collectively suggest that CREF enhances bone formation while simultaneously inhibiting bone resorption, creating an optimal healing environment likely due to its mechanical stability, improved periosteal blood supply, and reduced soft tissue trauma.

Inflammation is pivotal in fracture healing, but excessive or prolonged inflammatory responses can hinder bone regeneration and delay recovery [22]. C-reactive protein (CRP) is an acute-phase protein marker of systemic inflammation, with elevated levels correlating with tissue damage and delayed healing [23]. In this study, CRP levels were significantly lower in CREFG at 3d, 7d, and 14d postoperatively (*P*<0.05), suggesting that external fixation reduces soft tissue trauma and systemic inflammation. Interleukin-6 (IL-6) plays a dual role in fracture healing, promoting early inflammation and supporting bone repair in later stages [24]. Excessive IL-6 levels, however, may prolong inflammation and impair healing. Our results demonstrated significantly lower IL-6 levels in CREFG at all postoperative time points (*P*<0.05), indicating a more controlled inflammatoryresponse that may contribute to improved bone regeneration. Interleukin-1β (IL-1β) is a pro-inflammatory cytokine that stimulates immune responses and osteoclast activity [25]. Overexpression of IL-1β can lead to excessive bone resorption and delayed healing. In this study, IL-1β levels were markedly lower in CREFG than CRIFG (*P*<0.05), suggesting that CREF minimises unnecessary inflammatory activity, promoting faster recovery. Tumor necrosis factora-α (TNF-α) plays a complex role in bone metabolism, influencing osteoblast and osteoclast activity. WhileTNF-α can stimulate bone formation in early fracture healing, prolonged elevation may increase bone resorption and impair healing [26] [27]. Our findings demonstrated significantly lower TNF-α levels in CREFG postoperatively (*P*<0.05), further supporting the anti-inflammatory benefits of external fixation. By modulating the inflammatory response effectively, CREF appears to create a balanced healing environment, reducing excessive immune activation and allowing necessary healing processes.

Postoperative complications can significantly impact recovery and long-term functional outcomes.In this study, the incidence of adverse reactions (AR), including wound infection, chronic oedema, nonunion, nerve damage, and secondary fractures, was significantly lower in CREFG compared to CRIFG (18.52% vs 31.71%, *P*<0.05). This finding aligns with previous research suggesting that external fixation helps preserve soft tissue integrity, reduces deep infection risks, and promotes early mobilisation [28] [29]. Patients in CREFG also reported significantly lower Visual Analogue Scale (VAS) scores, reflecting reduced pain levels at multiple time points. The pain reduction observed with CREF may be attributed to its minimally invasive approach, which avoids extensive periosteal stripping and reduces intraoperative trauma. Furthermore, the higher Generic Quality of Life Inventory-74 (GQOLI-74) scores across all dimensions in CREFG suggest that external fixationenhances postoperative quality of life by facilitating earlier rehabilitation and minimising complications.

Despite these promising findings, this study has certain limitations. The retrospective design may introduce selection bias, and the study primarily focused on short-term outcomes. Long-term follow-up data on functional recovery and potential complications were not assessed. Future studies should include larger sample sizes and extended follow-up periods to evaluate the long-term durability of CREF’s benefits. Additionally, molecular investigations into the specific mechanisms by which CREF influences bone metabolism and inflammatory responses could provide further insights into its therapeutic potential [30].

Our findings suggest that CREF is superior to CRIF in treating tibiofibular fractures by enhancing bone metabolism, reducing inflammatory responses, alleviating pain, and improving postoperative quality of life. By minimising intraoperative trauma, preserving periosteal blood supply, and promoting early mobilisation, CREF represents an effective alternative for fracture management. Further prospective studies are needed to confirm these results and explore the long-term implications of external fixation in orthopaedic trauma care.

## Dodatak

### Ethics statement

This retrospective chart review study involving human participants was conducted according to the ethical standards of the institutional and national research committee, along with the 1964 Helsinki Declaration and its later amendments or comparable ethical standards. This study obtained approval from the Happy Center Hospital Shanghai Fifth People’s Hospital ethics committee.

### Availability of data and materials

All data generated and analysed during this study are included in this published article.

### Conflict of interest statement

All the authors declare that they have no conflict of interest in this work.
